# Simple method for large-scale production of macrophage activating factor GcMAF

**DOI:** 10.1038/s41598-020-75571-y

**Published:** 2020-11-05

**Authors:** Yoko Nabeshima, Chiaki Abe, Takeshi Kawauchi, Tomoko Hiroi, Yoshihiro Uto, Yo-ichi Nabeshima

**Affiliations:** 1 Laboratory of Molecular Life Science, Center of Biomedical Research and Innovation, Foundation for Biomedical Research and Innovation at Kobe, 2-2 Minatojima-Minamimachi Chuo-ku, Kobe, 650-0047 Japan; 2 Graduate School of Technology, Industrial and Social Science, Tokushima University, Tokushima, 770-8506 Japan

**Keywords:** Biochemistry, Biotechnology, Molecular biology

## Abstract

Human group-specific component protein (Gc protein) is a multifunctional serum protein which has three common allelic variants, Gc1F, Gc1S and Gc2 in humans. Gc1 contains an O-linked trisaccharide [sialic acid-galactose-*N*-acetylgalactosamine (GalNAc)] on the threonine^420^ (Thr^420^) residue and can be converted to a potent macrophage activating factor (GcMAF) by selective removal of sialic acid and galactose, leaving GalNAc at Thr^420^. In contrast, Gc2 is not glycosylated. GcMAF is considered a promising candidate for immunotherapy and antiangiogenic therapy of cancers and has attracted great interest, but it remains difficult to compare findings among research groups because different procedures have been used to prepare GcMAF. Here, we present a simple, practical method to prepare high-quality GcMAF by overexpressing Gc-protein in a serum-free suspension culture of ExpiCHO-S cells, without the need for a de-glycosylation step. We believe this protocol is suitable for large-scale production of GcMAF for functional analysis and clinical testing.

## Introduction

Human group-specific component protein (Gc), also known as Gc globulin (GcG) or vitamin D binding protein (DBP), is a 458-amino acid protein with an unmodified mass of 51.2 kDa. Gc protein is mainly synthesized in liver and is present at a high level in blood/plasma (300–600 mg/L), and at lower concentrations in colostrum and milk^1,2^. It was first described in 1959^3^, and three common allelic variants, Gc1F, Gc1S and Gc2 were subsequently identified in the human population. Relative to the Gc1F protein, Gc1S contains a glutamate residue in place of aspartate at position 416 (D416E mutation), while Gc2 contains a lysine residue in place of threonine at position 420 (T420K mutation)^4–6^. Gc protein has at least four distinct molecular functions. Namely, (1) Gc protein contains a single vitamin D-binding site at N-terminal domain 1, enabling it to work as a carrier protein of vitamin D metabolites in blood^2,7–9^, (2) it has a role in the circulating actin scavenger system, preventing narrowing of small blood vessels by binding to monomeric actin (G-actin) released from damaged cells in the events of cell injury and lysis^10,11^, (3) it is a precursor of a potent activator of macrophages (GcMAF, Gc-derived macrophage activating factor) and osteoclasts^12–15^, and (4) it serves as a chemotactic cofactor for C5a by interacting with cell-surface proteins of neutrophils^16,17^.

**Figure 1:**
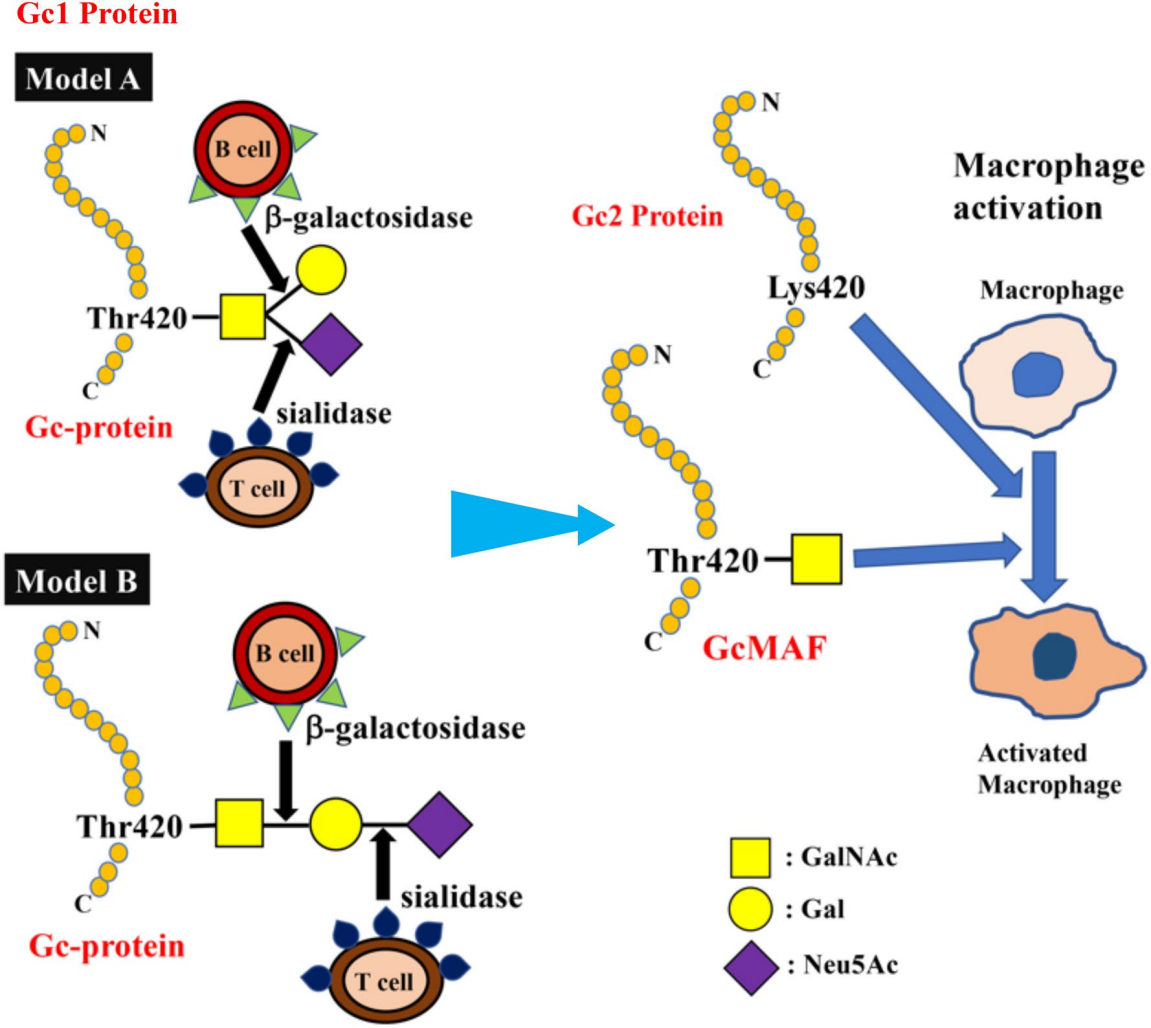
Structure of Gc protein and its conversion to GcMAF. Yamamoto et al. proposed (**Model A**) for the structure of DBP/Gc1F protein and its conversion to GcMAF^25^. In this model, GalNAc is covalently bound to Thr^420^ of the Gc1F protein and galactose and sialic acid are bound to GalNAc in a Y-branched arrangement. Therefore, removal of galactose and sialic acid exposes the GalNAc moiety and leads to the formation of activated GcMAF. However, Ravnsborg et al. recently proposed a linear model (**Model B**) based on mass spectrometry findings^23^. In this model, the three sugar moieties attached to threonine 420 are arranged in a linear fashion with GalNAc covalently bound to threonine, and galactose and sialic acid attached to the GalNAc in this order. Non-glycosylated Gc2 is also illustrated based on current mass spectrometry findings.

The C-terminal end of Gc1 (domain III) harbors a single glycosylation site. The carbohydrate structure was firstly elucidated by analysis of the products generated by treatment with several glycolytic enzymes^12,18,19^, and it was reported that Gc1 contains an O-linked trisaccharide with GalNAc attached to Thr^420^, followed by a galactose moiety, and a sialic acid (in Gc1F) or mannose moiety (in Gc1S). Gc2, which lacks this threonine residue, was thought to have only a disaccharide moiety composed of GalNAc and galactose, although more than 90% of Gc2 is present as a non-glycosylated form in humans^12,18,19^. However, current detailed glycan structural analyses using glycosidase treatment and mass spectrometry^6,20–22^ indicated that (1) Gc1F and Gc1S proteins have the same linear trisaccharide, sialic acid-galactose-GalNAc, on the Thr^420^ residue (Fig. 1 Model B)^23^ and (2) substitution of a lysine residue at the position corresponding to Thr^420^ in Gc2 prevents this isoform from being glycosylated at that position, and thus Gc2 is not glycosylated (Fig. 1). These conclusions are supported by the finding that Gc1 from cancer patients contains the same trisaccharide as Gc1 from healthy volunteers, namely, sialic acid-galactose-GalNAc-Thr^420^^24^.

Gc proteins are converted into GcMAF via de-glycosylation; specifically, it has been hypothesized that inflammation results in selective hydrolysis of galactose and sialic acid of Gc1 proteins by β-galactosidase of stimulated B lymphocytes and sialidase of T lymphocytes^13,25,26^, leaving GalNAc covalently attached to the threonine residue (Fig. 1). Reported activities of GcMAF include macrophage activation^27^, anti-angiogenesis activity^28–30^, and antitumor activity^31–33^. Indeed, human blood-derived GcMAF has been reported to be effective against metastatic colorectal, metastatic breast and prostate cancers^34–36^. The medication of GcMAF is, therefore, potential options for immunotherapy and antiangiogenic therapy against cancers. However, biological studies and clinical trials of GcMAF have yielded inconsistent results, probably because most laboratories and cancer clinics have produced their own GcMAFs using different procedures, even though all are based on sequential de-glycosylation of Gc proteins prepared from human blood. Thus, there is a need for methodology to provide a consistent product on a large scale for further studies.

Here, we present a practical method to prepare high-quality GcMAF by overexpressing Gc protein in a serum-free suspension culture of ExpiCHO-S cells, without the need for a de-glycosylation step. The synthesized GcMAF can be purified by a single step of vitamin D affinity column chromatography. This simple methodology enables the production of large amounts of high-quality GcMAF, and should greatly advance functional analyses and further clinical evaluation of GcMAF.

## Results

### Expression of Gc1F in CHO cells

**Figure 2:**
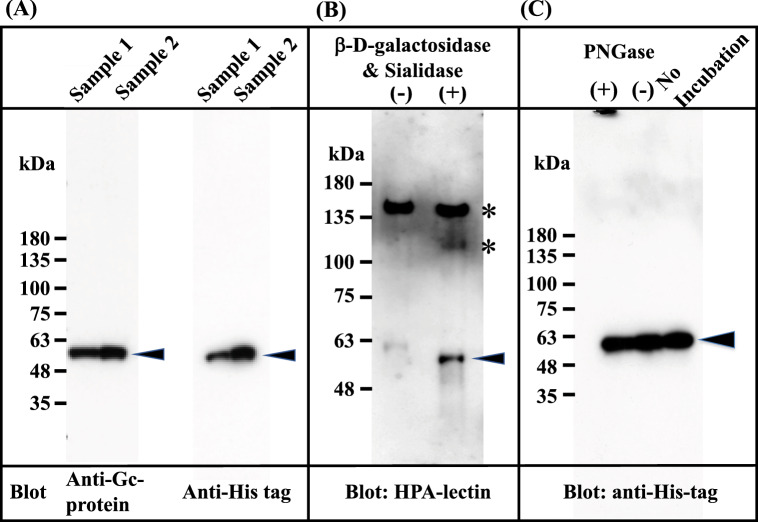
Characterization of Gc1F synthesized in CHO cells. Culture supernatants were adjusted to the composition and pH of the binding buffer of His Trap HP (Ni^2+^ pre-charged) column chromatography. The samples were applied to the column, which was washed and then eluted with elution buffer. The eluate was desalted by dialysis against 50 mM sodium phosphate pH 7.0 (desalted fraction). The desalted fraction was subjected to 5–20% SDS polyacrylamide gel and bands were blotted onto PVDF membrane. Anti-Gc antibody (**A**, left panel) and anti-His antibody (**A**; right panel) were used to detect His-tagged Gc1F and visualized by an enhanced chemiluminescence detection system. Aliquots of desalted His-tagged Gc1F (**B** (−)) and a sample treated with β-d-galactosidase and sialidase (**B** (+)) were characterized by HPA-lectin blotting. HPA-lectin-reactive Gc1F is indicated by an arrowhead (**B** (+)). Extra bands indicated by * are unknown HPA-lectin-reactive protein(s) that were removed during vitamin D affinity column chromatography (see Fig. 4A). *N*-Glycosylation of Gc-1F synthesized in CHO cells was analyzed (**C**). Desalted His-tagged Gc1F ((**C**) no-incubation) was treated with PNGase (**C** (+)) or incubated without PNGase (**C** (−)) and subjected to SDS gel electrophoresis. Full-length blots are included in a Supplementary Fig. 4.

The Gc1F-His expression vector (pcDNA3.4-TOPO^Gc1F-His^) (Supplementary Figs. 1 and 2) was transfected into CHO cells using lipofectamine 2000, and the cells were cultured for 3–4 days. The supernatant was applied to a His Trap HP column, which was washed with binding buffer and eluted with elution buffer solution. The eluate was desalted by dialysis against 50 mM sodium phosphate-buffered saline pH 7.4, and an aliquot of the desalted fraction was de-glycosylated with β-d-galactosidase and sialidase. These preparations were subjected to SDS gel electrophoresis, transferred to a membrane, and stained with anti-human Gc antibody, anti-His antibody and *biotin-conjugated Helix promatia* (HPA) lectin which roughly reacts to GalNAc. As expected, the expressed protein in the desalted fraction was detected with the antibodies against Gc and His (Fig. 2A), but not with biotin-conjugated HPA lectin (Fig. 2B (−)). However, the protein in the de-glycosylated fraction (Fig. 2B (+))was detected with HPA lectin, suggesting that Gc1F-His protein bearing O-linked trisaccharide (i.e., Sialic acid-Galactose-GalNAc-Thr^420^)^20–23^ was synthesized in CHO cells, and that sialic acid and galactose were removed by the de-glycosylation treatment (Fig. 2B (+)). This result is consistent with reports that Gc protein prepared from human blood can be converted to HPA lectin-reactive GcMAF by treatment with β-d-galactosidase and sialidase^34–36^. To examine whether the protein synthesized in CHO cells is *N*-glycosylated, the desalted His-tagged Gc1F was treated with PNGase and subjected to SDS gel electrophoresis (Fig. 2C). The bands marked by arrowheads showed similar mobilities, suggesting that Gc protein synthesized in CHO cells was not *N*-glycosylated.

### Production of HPA lectin-reactive Gc proteins without a de-glycosylation step

**Figure 3:**
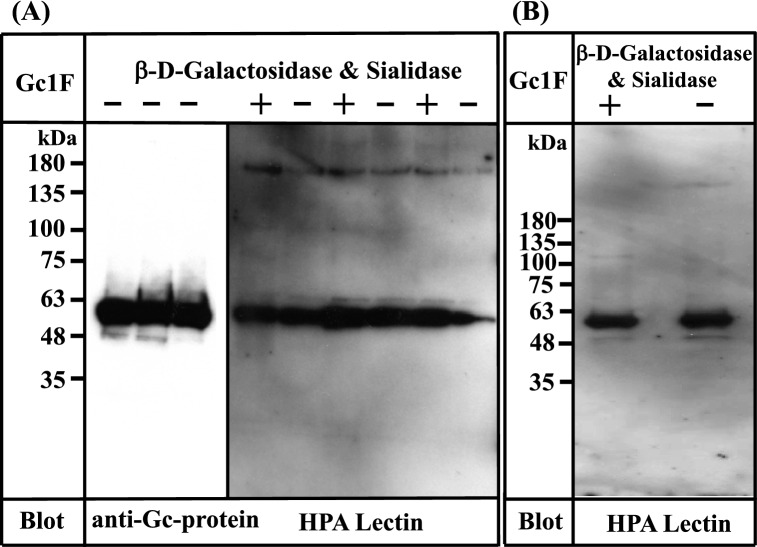
Characterization of Gc1F synthesized in ExpiCHO-S cells and in Expi293-F cells. (**A**) Three samples of Gc1F-His independently synthesized in ExpiCHO-S cells were characterized using anti-Gc antibody (left panel). Sugar modifications of His-tagged Gc1F alone (−) and after treatment with β-d-galactosidase and sialidase (+) were analyzed by biotin-conjugated HPA-lectin blotting (right panel). (**B**) Gc1F-His was synthesized in Expi293-F cells under serum-free suspension culture conditions. Sugar modifications of Gc1F-His alone (−) and after treatment with β-d-galactosidase and sialidase (+) were characterized by biotin-conjugated HPA-lectin blotting. Full-length blots are included in a Supplementary Fig. 4.

Based on the above observation, we attempted to establish a standard procedure for large-scale preparation of Gc-protein using serum-free suspension-cultured ExpiCHO-S cells, because this approach has been widely used for large-scale preparation of proteins. Briefly, ExpiCHO-S cells were pre-cultured in serum-free suspension culture medium for 2 days. Expression vector DNA (pcDNA3.4-TOPO^Gc1F-His^) (Supplementary Figs. 1 and 2) was transfected into the pre-cultured cells using Lipofectamine 2000 and culture was continued for 7–8 days; the survival rate of cells gradually decreased and fell below 90% (the recommended criterion to stop culture) at culture days 7 or 8. The culture supernatant was collected by centrifugation at 10,000 × *g* for 15 min, and applied to a Ni–NTA agarose column. The eluate was desalted by dialysis against phosphate buffer, and then de-glycosylated with β-d-galactosidase and sialidase. The de-salted and de-glycosylated fractions were each characterized by Western blot analysis (Fig. 3).

To our surprise, Gc1F-His expressed in ExpiCHO-S cells was detected not only with anti-Gc antibody (Fig. 3A, left panel), but also with HPA lectin, irrespective of treatment with or without de-glycosylation enzymes (Fig. 3A, right panel). Furthermore, the reactivity to HPA lectin was not further increased by de-glycosylation treatment (Fig. 3A, right panel), suggesting that HPA lectin-reactive saccharide (GalNAc-Thr^420^) was attached to almost all of the Gc1F-His synthesized in ExpiCHO-S cells under serum free suspension-culture conditions.

We next examined whether the above finding is specific to ExpiCHO-S cells. To test this, we synthesized GcIF-His in Expi293-F cells, another type of cells available for serum-free suspension culture. As shown in Fig. 3B, Gc1F-His expressed in Expi293-F cells was detected with both anti-Gc antibody (data not shown) and biotin-conjugated HPA lectin irrespective of treatment with or without de-glycosylation enzymes, suggesting that HPA lectin-reactive saccharide (GalNAc-Thr^420^) was present on the expressed protein.

**Figure 4:**
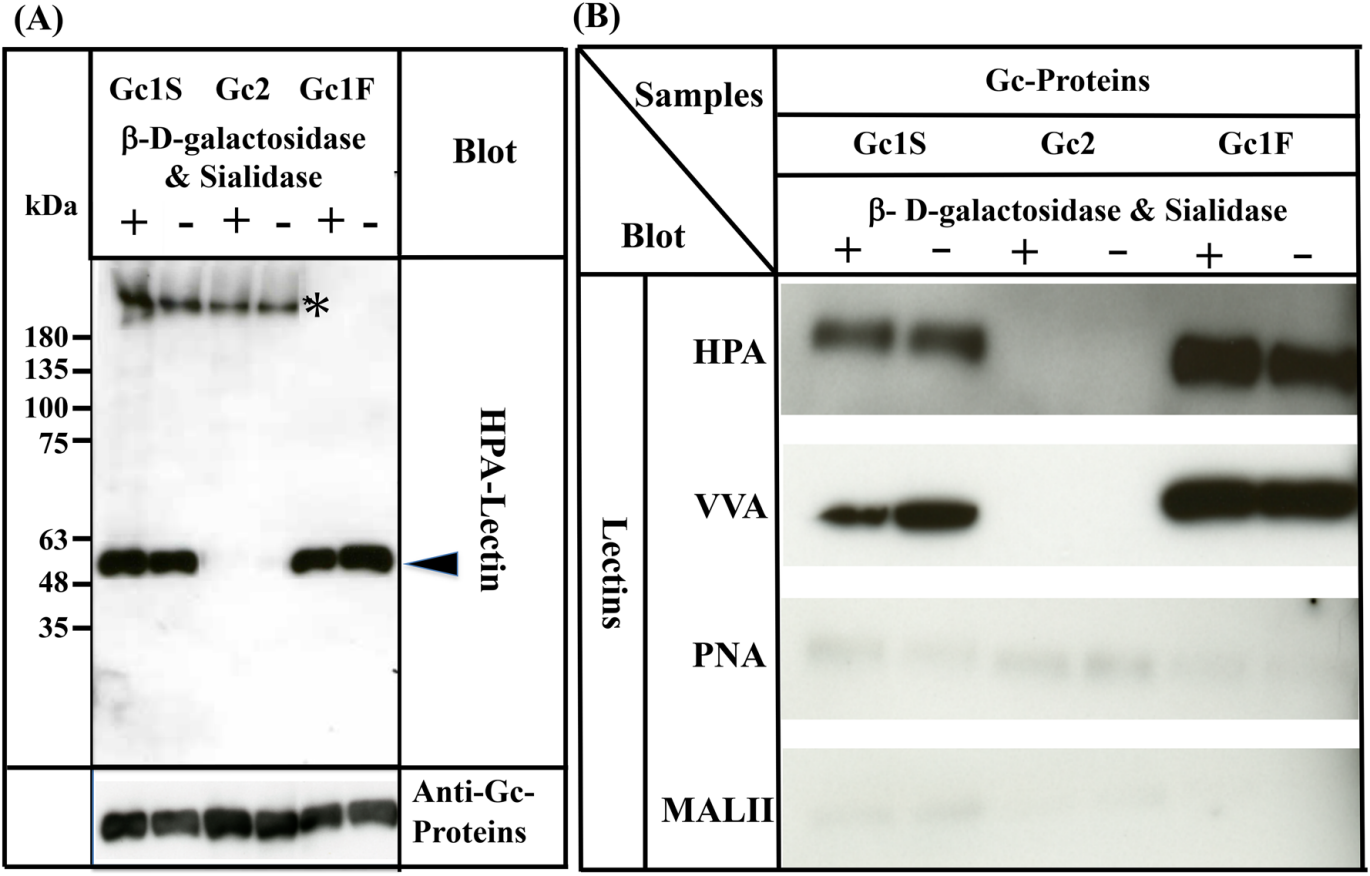
Characterization of Gc1S, Gc2 and Gc1F synthesized by ExpiCHO-S cells in serum-free suspension culture. Gc1S and Gc2 were synthesized by ExpiCHO-S cells transfected with pcDNA3.4-TOPO^Gc1S-His^ or pcDNA3.4-TOPO^Gc2-His^ (Supplementary Fig. 1) in serum-free suspension culture. Gc1S and Gc2 were purified by His Trap HP (Ni^2+^ pre-charged) column chromatography and desalted by dialysis against 50 mM sodium phosphate pH 7.0 (desalted fraction). Sugar moieties of the desalted fraction alone (−) and after treatment with β-d-galactosidase and sialidase (+) were analyzed by blotting with biotin-conjugated HPA-lectin (arrowhead). Purified Gc1F was analyzed as a control. Gc1S, Gc2 and Gc1F reacted with anti-Gc antibody (lower column). Extra bands indicated by * were unknown HPA-lectin reactive proteins that became undetectable after vitamin D affinity column chromatography (see lanes 1F+, 1F−). (**B**) Gc1F, Gc1S and Gc2 synthesized by ExpiCHO-S cells in serum-free suspension culture were purified by His Trap HP (Ni^2+^ pre-charged) column chromatography and Vitamin D affinity column chromatography, and then desalted by dialysis against 50 mM sodium phosphate pH 7.0 (desalted fraction). Sugar moieties of the desalted fraction without treatment (−) and after treatment with β-d-galactosidase and sialidase (+) were analyzed by blotting with HPA, VVA, PNA, or MALII lectins as indicated. Full-length blots are included in a Supplementary Fig. 4.

Since three allelic variants, Gc1F, Gc1S and Gc2, are found in humans^4–6^, we next examined whether HPA lectin-reactive forms of Gc1S and Gc2 are also synthesized under serum-free suspension culture conditions, by transfecting pcDNA3.4-TOPO^Gc1S-His^ and pcDNA3.4-TOPO^Gc2-His^ (Supplementary Fig. 1) into ExpiCHO-S cells. As shown in Fig. 4A, Gc1S-His was detected with biotin-conjugated HPA lectin, irrespective of treatment with or without de-glycosylation enzymes. However, Gc2-His was not detected by HPA lectin even after treatment with de-glycosylation enzymes.

Since many lectins only display moderate specificity, we confirmed the above results by using other lectins: biotinylated *Vicia villosa* (VVA) lectin, biotinylated *Maackia amurensis* (MAL-II) lectin II and biotinylated peanut (PNA) lectin. As expected, Gc1F and Gc1S reacted strongly with HPA and VVA lectins, which respond to GalNAc/Tn, but scarcely reacted with PNA and MALII lectins, which show a preference for Gal-GalNAc/T disaccharide and sialic acid/STn/ST, respectively (Fig. 4B). The reactions of Gc2 with HPA, VVA, PNA, and MALII lectins were negligible (Fig. 4B). Taken together, these results may indicate that Gc1F and Gc1S produced in ExpiCHO-S cells are glycosylated with GalNAc at Thr^420^, while Gc2 is not glycosylated.

### Purification of Gc proteins by vitamin D affinity column chromatography

**Figure 5:**
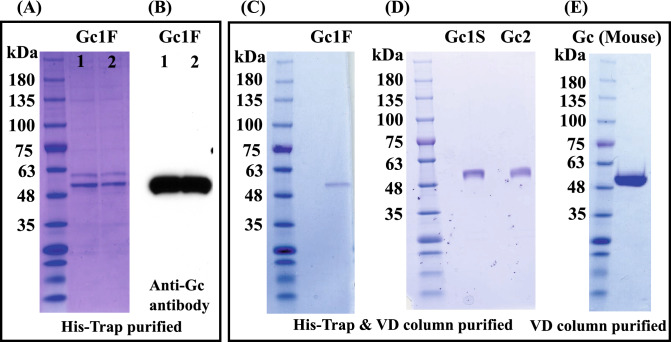
Purification of Gc proteins by vitamin D affinity column chromatography. CBB staining patterns of two independently prepared samples of Gc1F, purified with His-Trap column chromatography (**A**, Gc1F 1 and 2) and blotting patterns with anti-Gc antibody (**B**, Gc1F 1 and 2). The eluate from the His-Trap column (**A**, Gc1F 1) was subjected to vitamin D affinity column chromatography. Affinity-purified Gc1F was detected by CBB staining (**C**). Gc1S and Gc2 were similarly purified and stained with CBB (**D**). The CBB staining pattern of mouse GcMAF purified by a single step of vitamin D affinity column chromatography (**E**). Full-length gels and blots are included in a Supplementary Fig. 4.

Gc1 and Gc2 proteins synthesized in ExpiCHO-S cells were purified by His-tag column chromatography and vitamin D affinity column chromatography (Fig. 5A–D). The Gc1 and Gc2 proteins were each purified as a single band of 53,000 Da. We next tested whether the His-tag column chromatography step could be skipped; if so, it should be feasible to produce native Gc protein without the extra tag sequence. To examine this, we synthesized tag-less mouse Gc protein (Supplementary Fig. 1C, pcDNA3.4-TOPO^mouse-Gc^) in ExpiCHO-S cells and purified the product by a single step of vitamin D affinity column chromatography. The product was detected as a single band of 52,000 Da (Fig. 5E) and confirmed to be HPA lectin-reactive.

### Phagocytosis assay of Gc proteins synthesized in ExpiCHO-S cells

**Figure 6:**
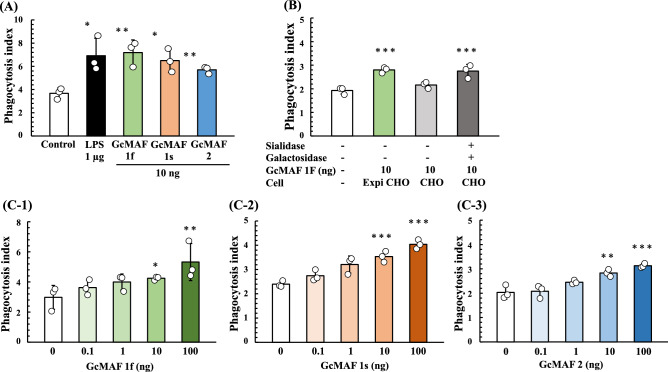
Phagocytosis assay of Gc proteins synthesized in ExpiCHO-S cells. Phagocytosis activation activity of vitamin D affinity column-purified GcMAF was analyzed. U937 cells were grown in RPMI-1640 medium and differentiated into macrophage-like cells by means of TPA treatment. (**A**) Differentiated U937 cells were exposed to Gc1F, Gc1S and Gc2 proteins for 3 h, and then Dynabeads were added to evaluate phagocytotic activity (average phagocytosis index: API). LPS (1 µg) was used as a positive control. (**B**) APIs of Gc-1F from ExpiCHO-S with a truncated structure (GalNAc-Thr^420^ type, positive control), Gc-1F from CHO with an elongated glycoform [Trisaccharide-Thr^420^ type, β-d-galactosidase (−) and Sialidase (−)] and Gc-1F from CHO with a truncated glycoform [GalNAc-Thr^420^ type, β-d-galactosidase (+) and sialidase (+)]. (**C**) Dose–response relationships for API of Gc1F (**C-1**), Gc1S (**C-2**) and Gc2 (**C-3**) from ExpiCHO-S. Values are mean ± standard deviation. The statistical significance of differences between control and treatment groups was determined by applying Dunnett's test (*p < 0.05, **p < 0.01).

We next analyzed the phagocytosis activation activity of Gc proteins synthesized in ExpiCHO-S cells. U937 cells were grown in RPMI-1640 medium and differentiated into macrophage-like cells by means of TPA treatment. Then, U937 cell-derived macrophages were activated by exposure to purified Gc proteins and LPS (a typical macrophage-activating chemical). Since the phagocytic activity of macrophages induced by Gc1F derived GcMAF is significantly increased at the concentration of 10 ng/ml^37^, we evaluated the average phagocytosis index (API) of the Gc proteins at this concentration. As shown in Fig. 6A, API was increased by the addition of Gc proteins and the activation levels of Gc1F and Gc1S were equivalent with that of LPS, used as a positive control, though the activation level of Gc2 was a little lower. We next examined the effect of de-glycosylation by comparing the macrophage-activating activities of Gc-1F from ExpiCHO-S with a truncated structure (GalNAc-Thr^420^, Positive control), Gc-1F from CHO with an elongated glycoform (Trisaccharide-Thr^420^) and Gc-1F from CHO with a truncated glycoform (after de-glycosylation reaction, GalNAc-Thr^420^). As shown in Fig. 6B, the APIs of Gc-1F from ExpiCHO-S with a truncated structure and Gc-1F from CHO with a truncated glycoform were similarly increased, whereas the activation by Gc-1F from CHO with an elongated glycoform was negligible, suggesting that Gc-1F from CHO is activated by de-glycosylation. We further confirmed that API was dose-dependently increased up to 100 ng/ml for Gc1F, Gc1S and Gc2 (Fig. 6C-1, -2, -3). The actual values obtained in the macrophage activation assay are given in Supplementary Table 1.

## Discussion

In this work, we present a simple procedure for the large-scale preparation of the phagocytosis-activation active form of Gc, namely GcMAF. In humans, the Gc1F and Gc1S allelic variants are synthesized as a phagocytosis-activation inactive and HPA lectin nonreactive forms in the liver, and are converted to phagocytosis-activation active and HPA lectin reactive forms by cleavage of the O-linked sialic acid-galactose-GalNAc-Thr^420^ to afford the monosaccharide (GalNAc-Thr^420^). In accordance with this, we found that the phagocytosis-activation inactive and HPA lectin nonreactive form of Gc1F was synthesized in CHO cells and converted to phagocytosis-activation active and HPA-lectin reactive form by the action of de-glycosylation enzymes (β-d-galactosidase and sialidase) (Figs. 2B, 6C). However, to our surprise, phagocytosis-activation active and HPA lectin reactive Gc1F was directly synthesized in ExpiCHO-S cells and in Expi293-F cells under serum-free suspension culture conditions. Furthermore, Gc1S and Gc2 were also synthesized as phagocytosis-activation active forms in ExpiCHO-S cells (Fig. 6). As expected, Gc1F and Gc1S were reactive with HPA and VVA-lectins. However, Gc2 (T420K mutation) was not. This suggests that (1) HPA-lectin-reactive saccharide may be not essential for the phagocytosis-activation activity of Gc2 protein, at least in vitro, and (2) if so, Gc2 synthesized in ExpiCHO-S cells may act as a phagocytosis-activation factor via a mechanism not involving GalNAc. This may support the hypothesis that the presence or absence of GalNAc at Thr^420^ of Gc protein is irrelevant in determining immune competency and/or cancer risk^38^, and may also be consistent with the fact that the risk of cancer in Gc2-2 homozygotes is decreased rather than increased, even though they are unable to produce GcMAF containing GalNAc-Thr^420^^39^.

A stereo view of the overall fold structure of human Gc protein^40^. indicates that amino acids 418–420, including the glycosylation site, are located in the vicinity of the loop-helix (H3) structure, and the sugar moiety seems to be located on the exterior of a globular conformation of H3 (Supplementary Fig. 3)^41^. Thus, the truncated sugar moiety (GalNAc-Thr^420^) could be recognized by a putative receptor protein on the cell surface of macrophages and might initiate complex formation between Gc1 protein and the putative receptor (Supplementary Fig. 3B). However, the recognition/interaction between the sugar moiety and putative receptor alone might be not sufficient for functional/stable complex formation and protein–protein interaction between Gc1 protein and the putative receptor would be required for the formation of a functional/stable complex to transduce the macrophage activation signal (Supplementary Fig. 3B). Thus, in this system, GalNAc-Thr^420^ may function as a marker for molecular recognition by the putative receptor, triggering the protein–protein interaction. In contrast, the trisaccharide attached to Thr^420^ is not recognized by the putative receptor and thus cannot initiate protein–protein interaction. Furthermore, the trisaccharide attached to Thr^420^ may interfere with the interaction between Gc1 protein and the putative receptor (Supplementary Fig. 3A). This might be the reason why the truncation of sialic acid and galactose is required for the conversion of inactive Gc protein to active GcMAF. Since Gc2 has no sugar chain, such interference with protein–protein interaction between Gc2 and the putative receptor protein would not occur (Supplementary Fig. 3C). Although it is still unclear how the putative receptor can recognize Gc2 without GalNAc on Thr^420^, the amino acid substitution (T420K mutation) in Gc2 might favor interaction with the putative receptor and if so, Gc2 itself might be always functional, without activation. Such a non-regulated interaction between Gc2 and the putative receptor may explain why the risk of cancer in Gc2-2 homozygotes is decreased rather than increased^39^. Thus, our results provide an intriguing clue to the possible role(s) of the glycan moiety in macrophage activation signaling.

Our present data indicate that Gc1 protein synthesized in ExpiCHO-S cells is almost entirely HPA-lectin-reactive, suggesting that the newly synthesized Gc1 bears only the monosaccharide (GalNAc-Thr^420^), and the following extension reactions of the *O*-glycan do not occur. Although the underlying mechanism of production of this truncated *O*-glycan is unknown, it seems likely that the step-by-step glycosylation process in the Golgi apparatus is altered in ExpiCHO-S cells. Several tumor cell lines show altered distribution or partial loss of *O*-glycosylation enzymes in the Golgi apparatus^42^, and alteration of the *O*-glycan biosynthesis pathway(s) might occur similarly in ExpiCHO cells. Alternatively, Gc1 protein might undergo premature exit from an early compartment of the Golgi apparatus. Thus, our results provide an interesting future direction for further studies of the machinery of protein glycosylation and secretion.

In conclusion, we established methodology to produce biologically active GcMAF in large quantities by overexpressing Gc proteins in ExpiCHO-S cells under serum-free suspension culture conditions. Notably, the synthesized GcMAF can be purified in a single step by means of vitamin D affinity column chromatography. This simple protocol is expected to be suitable for large-scale production of high-quality GcMAF for functional analysis and clinical testing.

## Materials and methods

### Construction of Gc-protein expression vectors

We obtained a double-stranded DNA encoding a fusion protein consisting of human Gc1F protein and a histidine tag (His) at the C-terminal site (Gc1F-His DNA) by using the Oligo-DNA production service of Invitrogen (Supplementary Fig. 1A). Some triplet codons were modified to accord with the codon usage rates in animal cells in order to maximize the efficiency of translation. Gc1F-His DNA was inserted into the TOPO cloning site of pcDNA3.4-TOPO Vector (Invitrogen) (termed pcDNA3.4-TOPO^Gc1F-His^), in which the inserted Gc1F-His DNA is transcribed under the control of CMV promoter (Supplementary Fig. 2). To design Gc1S-His DNA, the GAT codon for aspartic acid^416^ (D^416^) was replaced with the GAG codon for glutamic acid (E^416^) (termed pcDNA3.4-TOPO^Gc1S-His^, Supplementary Fig. 1B-1). To design Gc2-His DNA, the ACC codon for threonine^420^ (T^420^) was replaced with the AAG codon for lysine (K^420^) (termed pcDNA 3.4-TOPO^Gc2-His^) (Supplementary Fig. 1B-2)^4–6^. We also synthesized a mouse Gc expression plasmid (pcDNA3.4-TOPO^mouse-Gc^) (Supplementary Fig. 1C). Large amounts of these expression vector DNAs were prepared by using an EndoFree Plasmid Kit (QIAGEN 12362) according to the supplier’s protocol.

### CHO cell culture and transfection of Gc1F-His expression plasmid

Chinese hamster ovary cells (CHO) were cultured in F12 medium (GIBCO 11765) supplemented with 10% heat-inactivated fetal bovine serum (FBS, Biowest S1820), 100 U/ml penicillin, and 100 μg/ml streptomycin (Sigma-Aldrich P4333) at 37 °C in a humidified atmosphere of 5% CO_2_. Cells were cultured to approximately 80% confluence in 6-well culture plates. The pcDNA3.4-TOPO^Gc1F-His^ expression plasmid (2.5 μg) and Lipofectamine 2000 (15 μl) (ThermoFisher Scientific Co.) were added in 2 ml medium per well. The plates were incubated for 3 days, then the culture media and cells were harvested. The expression level and sugar modification of Gc1F-His were evaluated.

### Culture of ExpiCHO-S and Expi293-F cells

ExpiCHO-S cells (ThermoFisher Scientific Co. ExpiCHO-S Cells, Catalog number: A29127) were suspension-cultured in 25 ml of serum-free ExpiCHO Expression medium in an incubator at 37 °C with a humidified atmosphere of 8% CO_2_ in air on an orbital shaker platform rotating at 125 rpm to a final density of 6 × 10^6^ viable cells/ml. A Gc expression plasmid (15 μg) (pcDNA3.4-TOPO^Gc1F-His^, pcDNA3.4-TOPO^Gc1S-His^, pcDNA3.4-TOPO^Gc2-His^ or pcDNA3.4-TOPO^mouse Gc^) was diluted with 1.0 ml of Opti PRO SFM (ThermoFisher Scientific Co.) and mixed with 80 μl of ExpiFectamine CHO Reagent (ThermoFisher Scientific Co.), previously diluted with 920 μl of Opti PRO SFM. This mixture was incubated for 5 min at room temperature and added to the ExpiCHO-S cell culture flask. The cells were incubated overnight, then 150 μl of ExpiCHO Enhancer and 6 ml of ExpiCHO Feed (ThermoFisher Scientific Co.) were added, and suspension culture was continued for 6–8 days (according to the supplier’s protocol, cell culture can be continued for up to 14 or 15 days). Then, we harvested the cells and media and analyzed the expression levels and sugar modifications of the Gc proteins.

Expi293F cells (ThermoFisher Scientific Co. Expi293F cells, Catalog number: A14528) were suspension-cultured in 30 ml of Expi293 Expression medium in an incubator at 37 °C with a humidified atmosphere of 8% CO_2_ in air on an orbital shaker platform rotating at 125 rpm to a level of 2.5 × 10^6^ cells/ml with > 95% viability. Gc expression plasmids (30 μg) were diluted with 1.5 ml of Opti-MEM (ThermoFisher Scientific Co.) and mixed with 80 μl of Expifectamine 293 reagent (ThermoFisher Scientific Co.), previously diluted with Opti-MEM to 1.5 ml. This mixture was incubated for 20 min at room temperature and then added to an Expi293-F cell culture flask. The cells were incubated under the conditions specified above and, after 18 h, 150 μl of Expifectamine 293 Transfection Enhancer 1 and 1.5 ml of Expifectamine 293 Transfection Enhancer 2 (ThermoFisher Scientific Co.) were added to the flask. The cells and the media were harvested at 3 days post-transfection. The expression levels and sugar modifications of the produced Gc proteins were characterized.

### Purification of Gc-His proteins with a His-Tag column

For the purification of histidine-tagged Gc proteins, we performed His Trap HP (Ni^2+^ pre-charged) column chromatography (GE Healthcare Life Sciences) according to the manufacturer’s protocol. Briefly, (1) the His Trap HP column (5 ml) was washed with binding buffer (20 mM sodium phosphate, 150 mM NaCl and 20 mM imidazole pH 7.4), (2) the cell culture supernatant was adjusted to the composition and pH of the binding buffer and filtered through a 0.45 μm filter, and (3) the filtrate was applied to the column, which was washed with binding buffer and eluted with elution buffer (20 mM sodium phosphate, 150 mM NaCl and 500 mM imidazole pH 7.4). Desalting was done by dialysis against 50 mM sodium phosphate pH 7.0.

### Assessment of cell proliferation and cell numbers

We determined the viable and total cell counts by means of the trypan-blue exclusion method. Cell suspensions were diluted with 0.5% trypan-blue solution and the cells were counted on a Neubauer hematocytometer.

### Quantitation of total protein

Protein concentrations were determined with BCA Protein Assay Kit (Thermo Fisher 23227) or by measuring the absorbance at 280 nm.

### Western blot analysis of Gc proteins and lectin blot for glycan moieties

The synthesized human Gc-His proteins were mixed with equal amounts of SDS sampling buffer (125 mM Tris–HCl pH 6.8, 4% SDS, 60% glycerol, 10 mM EDTA, 0.1 M DTT, 0.01% BPB, 10% 2-mercaptoethanol) and heated at 90 °C for 2 min. Samples were electrophoresed on 5–20% SDS polyacrylamide gel (Nacalai tesque, Kyoto, Japan) and bands were transferred to PVDF membranes (Merck Millipore Ltd., ME) using a semi-dry blotting device. The blot was incubated at 4 °C overnight with anti-Gc antibody (Abcam, ab153922) or with anti-His-tag antibody (Abcam ab18184), previously diluted in a blocking buffer containing 5% skim milk in TBST (25 mM Tris–HCl pH 7.4, 150 mM NaCl and 0.1% Tween 20). The primary antibodies, anti-DBP/Gc and anti-His-tag antibodies, were reacted with horseradish peroxidase (HRP)-conjugated anti-rabbit IgG (NA934, GE Healthcare) and anti-mouse IgG (GE Healthcare, NA9310v), respectively, and visualized by use of an enhanced chemiluminescence detection system (ECL prime, Amersham Bioscience). Biotin-conjugated *Helix pomatia* (HPA) lectin for Tn (Sigma-Aldrich, L6512) was used to detect the GalNAc/Tn moiety as previously reported^37^. In addition, biotin-conjugated *Vicia villosa* (VVA) lectin for GalNAc/Tn (Vector Laboratories, B-1235), biotin-conjugated *Maackia amurensis* lectin II (MAL-II) for sialic acid/ STn/ST (Vector Laboratories, B-1265) and biotin-conjugated peanut (PNA) lectin for Gal-GalNAc/T disaccharide (GeneTex, BTX01507) were used to confirm the glycosylation of Gc1 and Gc2 proteins.

### Purification of Gc proteins by vitamin d-sepharose column chromatography

The suspension-culture supernatant or His-trap column eluate was applied to a 25(OH)D_3_-sepharose column prepared according to Link et al.^43^. The column was washed with binding buffer (50 mM Tris–HCl, 1.5 mM EDTA, 150 mM NaCl and 0.1% Triton X-100 pH 7.4) and eluted with 6 M guanidine. For desalting, the eluate was dialyzed against 10 mM sodium phosphate pH 7.4.

### Phagocytosis assay

The phagocytosis activity of GcMAF was assayed according to the reported method^44^ with slight modifications. U937 cells (human myeloid leukemia cell line; KAC Co., Ltd., Ritto, Japan) were grown in RPMI-1640 medium (Gibco by Life Technologies, Grand Island, USA) supplemented with 10% (v/v) heat-inactivated FBS (Gibco Life Technologies, Grand Island, USA) and penicillin–streptomycin (Sigma-Aldrich, USA) in a fully humidified 5% CO_2_/95% air atmosphere at 37 °C. To induce differentiation into macrophage-like cells, 2.5 × 10^6^ U937 cells seeded onto 10 cm culture dishes were treated with 10 ng/ml 12-*O*-tetra-decanoylphorbol-13-acetate (TPA) (Nacalai tesque Co., Ltd., Kyoto, Japan) for 3 days, washed with 3 ml of Dulbecco’s PBS (Gibco Life Technologies Grand Island, USA) twice, and then trypsinized (1 ml of trypsin–EDTA per 10 cm dish) for 2–3 min at 37 °C. Then 2.5 × 10^5^ trypsinized cells were dispersed in 10% FBS/RPMI-1640 medium, layered onto cover-glasses in a 24-well plate, and treated with 10 ng/ml TPA for 3 days. These cells were incubated with serum-free RPMI-1640 medium for 15 h and pretreated with GcMAF for 3 h by replacing the medium with serum-free RPMI-1640 medium containing 10 ng/ml GcMAF^1f^, GcMAF^1s^, or GcMAF^2^. LPS (1 µg/ml) was used as a positive control. After 3 h pretreatment, the medium was replaced by serum-free RPMI-1640 medium containing 90 μg/ml protein G magnetic beads (Dynabeads Protein G; Invitrogen, Carlsbad, USA) and incubated for 1.5 h. The cover-glasses were washed with phosphate-buffered saline and the cells were fixed with methanol for 1 min. The fixed cells were air-dried, stained with Giemsa’s azur eosin methylene blue solution (Merck KGaA, Darmstadt, Germany) for 1 h and washed with distilled water. The cells were air-dried, and then immersed in Malinol (Muto Pure Chemicals Co., Ltd., Tokyo). Cells were imaged with an upright microscope (DM6B, Leica Microsystems, Germany); nine random areas in each cover-glass sample were examined. The activity of GcMAF was expressed as the average phagocytosis index (API), calculated according to the following formula: API = (number of internalized beads within the macrophages)/(number of macrophages within the photograph). We prepared three independent samples for each analysis and thus 27 areas (9 random areas × 3 independent samples) were analyzed for each sample. The above examination was repeated twice.

### Statistical analysis

All values are expressed as mean ± standard deviation. Statistical analyses were performed with EZR (Saitama Medical Center, Jichi Medical University, Saitama, Japan)^45^, which is a graphical user interface for R (The R Foundation for Statistical Computing, Vienna, Austria); specifically, it is a modified version of R commander designed to add statistical functions frequently used in biostatistics. The statistical significance of differences between control and treatment groups was determined by use of Dunnett’s test.

## Supplementary information

Supplementary Information.
