# Supplementary material for: Simple method for large-scale production of macrophage activating factor GcMAF
Source: Sci Rep. 2020 Nov 5;10:19122. doi: 10.1038/s41598-020-75571-y (PMC7645693; doi:10.1038/s41598-020-75571-y)
Supplement: Supplementary file 1 — Supplementary Information. [file 41598_2020_75571_MOESM1_ESM.pdf]

Simple method for large-scale production of macrophage activating factor GcMAF

Yoko Nabeshima[1], Chiaki Abe[1], Takeshi Kawauchi[1], Tomoko Hiroi[1], Yoshihiro Uto[2] and Yo-ichi Nabeshima[1]*

1. Laboratory of Molecular Life Science, Center of Biomedical Research and Innovation, Foundation for Biomedical Research and Innovation at Kobe 2-2 Minatojima-Minamimachi Chuo-ku, Kobe 650-0047 Japan

2. Graduate School of Technology, Industrial and Social Science, Tokushima University, Tokushima, 770-8506, Japan

*To whom all correspondence should be addressed (nabemr@lmls-kobe.org)

Supplementary Figure 1. DNA sequences encoding Gc1F, GcIS, Gc2 and mouse Gc proteins

The expression vector of human Gc1F-His (pcDNA3.4-TOPO$^{Gc1F\text{-}His}$) was prepared by using the Invitrogen GeneArt gene synthesis service (Thermo Fisher Scientific).

(A) Sequence of synthesized DNA encoding Gc1F-His:

1 *XbaI* *BamHI*

GGACCGATCCAGCCTCCGGACTCTAGAGGATCGAACCCTTGGATCCACCATGAAGCGGGT

Met

61

GCTGGTGCTGCTGCTGGCCGTGGCCTTTGGACACGCCCTGGAAAGAGGCCGGGACTACGA

121

GAAGAACAAAGTGTGCAAAGAGTTCAGCCACCTGGGCAAAGAGGACTTCACCAGCCTGAG

181 *BstBI* *PvuII*

CCTGGTGCTGTACAGCCGGAAGTTCCCCAGCGGCACCTTCGAACAGGTGTCCCAGCTGGT

NarI

241 *StuI* *KasI*

CAAAGAAGTGGTGTCCCTGACCGAGGCCTGTTGCGCCGAAGGCGCCGACCCTGACTGCTA

301

CGATACCAGAACAAGCGCCCTGAGCGCCAAGAGCTGCGAGAGCAACAGCCCCTTTCCTGT

361

GCACCCTGGCACCGCCGAGTGCTGCACAAAAGAGGGCCTGGAACGGAAGCTGTGCATGGC

421 *EcoRI*

CGCCCTGAAGCACCAGCCCCAGGAATTCCCTACCTACGTCGAGCCCACCAACGACGAGAT

481 *StuI*

TTGCGAGGCCTTCAGAAAGGACCCCAAAGAGTACGCCAACCAGTTCATGTGGGAGTACAG

541

CACCAACTACGAGCAGGCCCCCCTGAGCCTGCTGGTGTCCTACACCAAGAGCTACCTGAG

601 *PstI*

CATGGTCGGAAGCTGCTGCACCAGCGCCAGCCCTACCGTGTGCTTCCTGAAAGAGCGGCT

661 *PvuII*

GCAGCTGAAGCACCTGTCCCTGCTGACCACCCTGAGCAACAGAGTGTGCAGCCAGTACGC

721 *BclI*

CGCCTACGGCGAGAAGAAGTCCCGGCTGAGCAACCTGATCAAGCTGGCCCAGAAGGTGCC

781

CACCGCCGACCTGGAAGATGTGCTGCCTCTGGCCGAGGACATCACCAACATCCTGAGCAA

841

GTGCTGCGAGTCCGCCAGCGAGGACTGCATGGCCAAAGAGCTGCCCGAGCACACCGTGAA

901

GCTGTGCGACAACCTGAGCACCAAGAACAGCAAGTTCGAGGACTGCTGCCAGGAAAAGAC

961 *NcoI* *PvuII*

CGCCATGGACGTGTTCGTGTGCACCTACTTCATGCCTGCCGCCCAGCTGCCTGAGCTGCC

1021

AGATGTGCGGCTGCCCACCAACAAGGACGTGTGCGACCCCGGCAACACCAAAGTGATGGA

1081

CAAGTACACCTTCGAGCTGAGCCGGCGGACCCATCTGCCCGAAGTGTTTCTGTCCAAGGT

1141

GCTGGAACCCACCCTGAAGTCCCTGGGCGAGTGCTGCGACGTGGAAGATAGCACCACCTG

1201 ***ApaI***

TTTCAACGCCAAGGGCCCCCTGCTGAAGAAAGAGCTGAGCAGCTTCATCGACAAGGGCCA

1261

GGAACTGTGCGCCGACTACAGCGAGAACACCTTCACCGAGTACAAGAAGAAGCTGGCCGA

1321 ***HincII***

GCGGCTGAAGGCCAAGCTGCCTGATGCCACACCTACCGAGCTGGCCAAGCTGGTCAACAA

D416 A417 T418 P419 T420

1381 ***PstI***

GCGGAGCGACTTCGCCAGCAACTGCTGCAGCATCAACAGCCCCCCACTGTACTGCGACAG

1441

CGAGATCGACGCCGAGCTGAAGAACATCCTGCACCACCACCATCACCATCATCACCACCA

H H H H H H H H H H

1501 *EagI* *Age I* ***EcoRV***

TTGATGAGCGGCCGCAAGGGTTCGATCCCTACCGGTTAGTAATGAGTTTGATATC

Stop Stop

(B) To obtain the expression vectors for Gc1S (Glu^{416}) and Gc2 (Lys^{420}), we synthesized the following two DNA fragments by using Invitrogen's GeneArt Strings System. These DNA

fragments were digested with ApaI and EcoRV, and then ligated into the ApaI-EcoRV sites of pcDNA3.4-TOPO$^{Gc1F\text{-}His}$ to introduce a D416E mutation (for Gc1S) and a T420K mutation (for Gc2).

(B-1) Sequence encoding Gc1S (Asp^{416} to Glu^{416}, highlighted in green):

1201 ***ApaI***

TTTCAACGCCAAGGGCCCCCTGCTGAAGAAAGAGCTGAGCAGCTTCATCGACAAGGGCCA

1261

GGAACTGTGCGCCGACTACAGCGAGAACACCTTCACCGAGTACAAGAAGAAGCTGGCCGA

1321 ***HincII***

GCGGCTGAAGGCCAAGCTGCCTGAGGCCACACCTACCGAGCTGGCCAAGCTGGTCAACAA

E^{416} A^{417} T^{418} P^{419} T^{420}

1381 ***PstI***

GCGGAGCGACTTCGCCAGCAACTGCTGCAGCATCAACAGCCCCCCACTGTACTGCGACAG

1441

CGAGATCGACGCCGAGCTGAAGAACATCCTGCACCACCACCATCACCATCATCACCACCA

1501 ***EcoRV***

TTGATGAGCGGCCGCAAGGGTTCGATCGGTACCGGTTAGTAATGAGTTTGATATCGCACGC

Stop Stop

(B-2) Sequence encoding Gc2 (Thr^{420} to Lys^{420}, highlighted in green):

1201 ***ApaI***

TTTCAACGCCAAGGGCCCCCTGCTGAAGAAAGAGCTGAGCAGCTTCATCGACAAGGGCCA

1261

GGAACTGTGCGCCGACTACAGCGAGAACACCTTCACCGAGTACAAGAAGAAGCTGGCCGA

1321 ***HincII***

GCGGCTGAAGGCCAAGCTGCCTGATGCCACACCTAAGGAGCTGGCCAAGCTGGTCAACAA

D^{416} A^{417} T^{418} P^{419} K^{420}

1381 ***PstI***

GCGGAGCGACTTCGCCAGCAACTGCTGCAGCATCAACAGCCCCCCACTGTACTGCGACAG

1441

CGAGATCGACGCCGAGCTGAAGAACATCCTGCACCACCACCATCACCATCATCACCACCA

1501 ***EcoRV***

TTGATGAGCGGCCGCAAGGGTTCGATCGGTACCGGTTAGTAATGAGTTTGATATCGCACGC.

StopStop

(C) The sequence encoding mouse Gc :

1 *XbaI* *BamHI*

GGACCGATCCAGCCTCCGGACTCTAGAGGATCGAACCCTTGGATCCACCATGAAGCGAGT

Met

61

GCTGGTTCTGCTGCTGGCCCTGGCTTTTGGACACGCTCTGGAAAGAGGCAGAGACTACGA

121

GAAGGACAAAGTGTGCAACGAGCTGGCCATGCTGGGCAAAGAGGACTTCAGAAGCCTGAG

181

CCTGATCCTGTACAGCAGAAAGTTCAGCAGCAGCACCTTCGAGCAAGTGAACCAGCTCGT

241 *NarI/KasI* *BspMI*

GAAAGAAGTGGTGTCCCTGACCGAGGAATGCTGTGCTGAAGGCGCCGATCCTACCTGCTA

301

CGACACCAGAACAAGCGAGCTGAGCGTGAAGTCCTGCGAGAGCGACGCTCCTTTTCCTGT

361 *XhoI*

GCACCCTGGCACACCTGAGTGCTGCACAAAAGAGGGCCTCGAGAGAAAGCTGTGCATGGC

421 *BglII*

CGCTCTGAGCCACCAGCCTCAAGAGTTCCCTACCTACGTGGAACCCACCAACGACGAGAT

481

CTGCGAGGCTTTCAGAAGGGACCCTAAGGGCTTCGCCGACCAGTTCCTGTACGAGTACAG

541

CAGCAACTACGGCCAGGCTCCTCTGCCACTGCTGGTGGCCTACACCAAGAACTACCTGAG

601 *PstI*

CATGGTCGGAAGCTGCTGCACCAGCGCTAACCCTACCGTGTGCTTTGTGAAAGAACGGCT

661

GCAGATGAAGCACCTGTCTCTGCTGACCACCATGAGCAACAGAGTGTGCTCTCAGTACGC

721 *BclI*

CGCCTACGGCAAAGAGAAGTCCAGACTGTCCCACCTGATCAAGCTGGCCCAGAAGGTGCC

781

AACCGCCAACCTGGAAAATGTGCTGCCTCTGGCCGAGGATTTCACCGAGATCCTGAGCAG

841 *BglII*

ATGCTGCGAGTCCACCAGCGAGGACTGTATGGCTTCTGAGCTGCCCGAGCACACCATCAA

901

GATCTGCCAGAACCTGAGCAAGAAGAACAGCAAGTTCGAGGAATGTTGCCAAGAGAACAC

961 *PstI PvuII*

CCCGATGAACATCTTCATGTGCACCTACTTCATGCCTGCCGCCGAGCCTCTGCAGCTGCC

1021 *AccI* *NcoI*

TGCTATTAAGCTGCCTACCGGCAAGGACCTGTGCGGCCAGTCTACAACACAGGCCATGGA

1081

CCAGTACACCTTTGAGCTGAGCAGACGGACCCAGGTGCCAGAGGTGTTCCTGTCCAAAGT

1141

GCTGGAACCTACACTCAAGACCCTGAGAGAGTGTTGCGACACCCAGGACAGCGTGGCCTG

1201 *PvuII*

TTTCTCTACACAGAGCCCTCTGCTGAAGAGACAGCTGACCAGCTTCATCGAGAAGGGCCA

1261

AGAGATGTGCGCCGACTACAGCGAGAACACCTTCACCGAGTACAAGAAGAAGCTGGCCGA

1321

GAGACTGAGGACCAAGACACCCAACACAAGCCCTGCCGAGCTGAAGGACATGGTGGAAAA

1381 *PstI*

GCACAGCGACTTCGCCAGCAAGTGCTGCTCCATCAACAGCCCTCCACTGTACTGCAGCAG

1441 *PstI* *EagI/ NotI*

CCAGATCGACGCCGAGATGATCGACACCCTGCAGTCCTAAGCGGCCGCAAGGGTTCGATC

Stop

Supplementary Figure 2. Structure of the Gc-protein expression plasmid

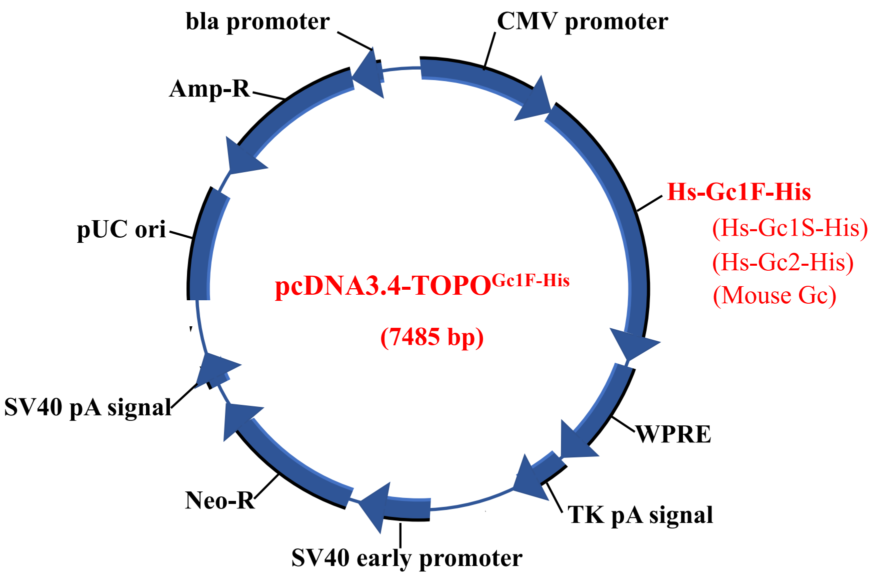

Supplementary Figure 3. Schematic representation of the interaction between GcMAF and the putative receptor

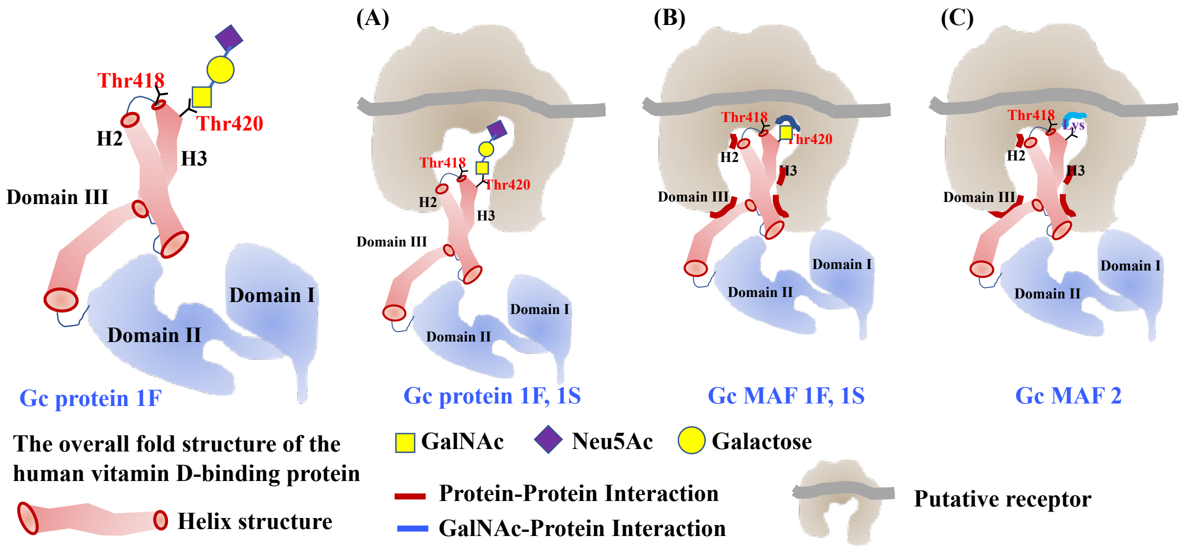

Supplementary Figure 4.

Photographs of full-length gels and blots of Fig.2 to Fig.5

Full length blots of Fig. 2(A) and 2(B)

Fig.2A **Fig.2B**

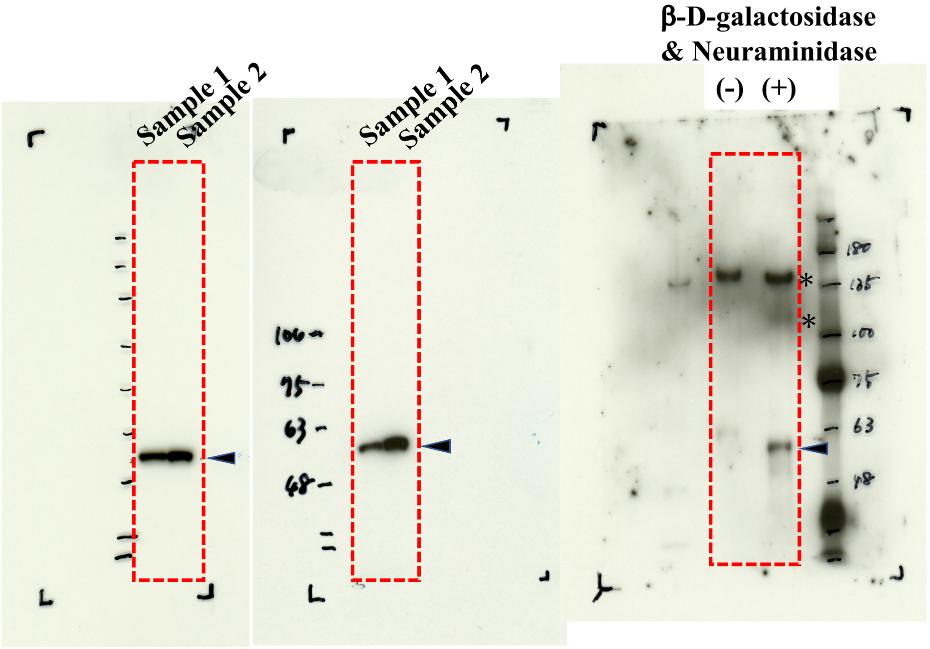

Anti-Gc-protein **Anti-His tag** **HPA-Lectin**

Full length blots of Fig. 2(C)

Fig.2C

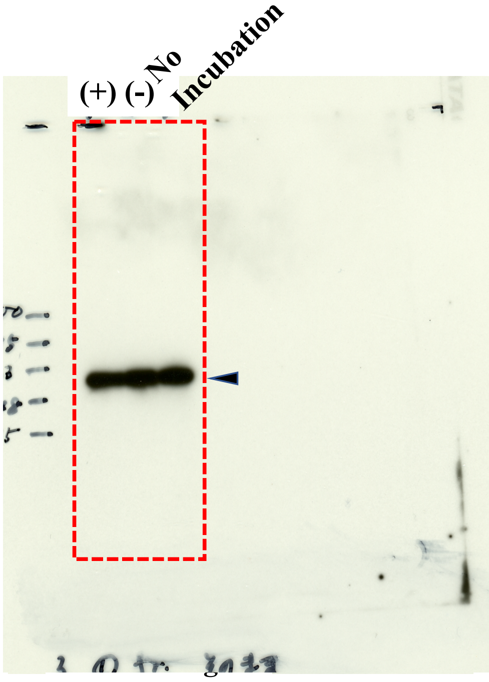

Anti-His tag

Full length blot of Fig.3 A

Fig3A left

Fig3A right

β-D-Galactosidase & Neuraminidase

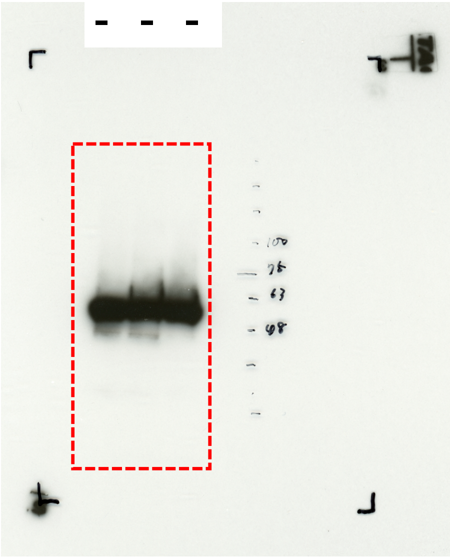

Anti-Gc-protein

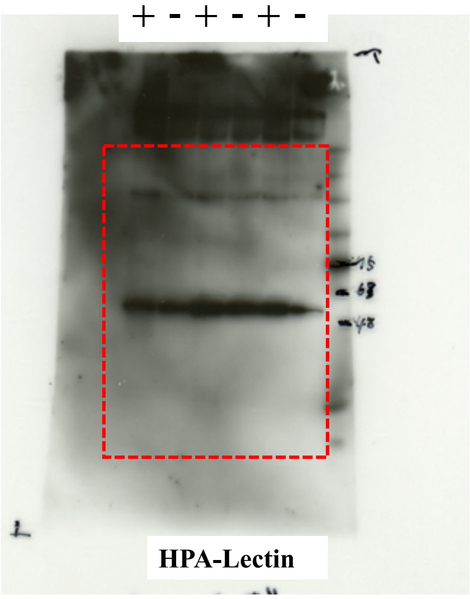

Full length blots of Fig. 3(B)

Fig3B

β-D-Galactosidase & Neuraminidase

\+ —

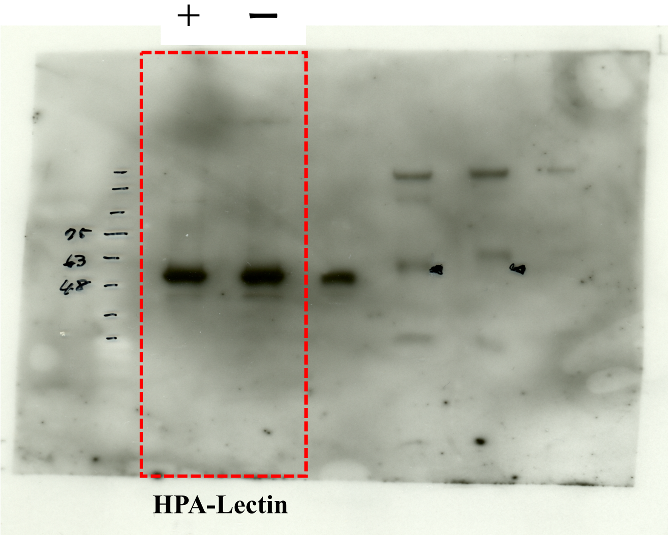

Full length blots of Fig. 4(A)

Fig4A upper Panel

Gc1S Gc2 Gc1f

β-D-galactosidase & Neuraminidase

\+ - + - + -

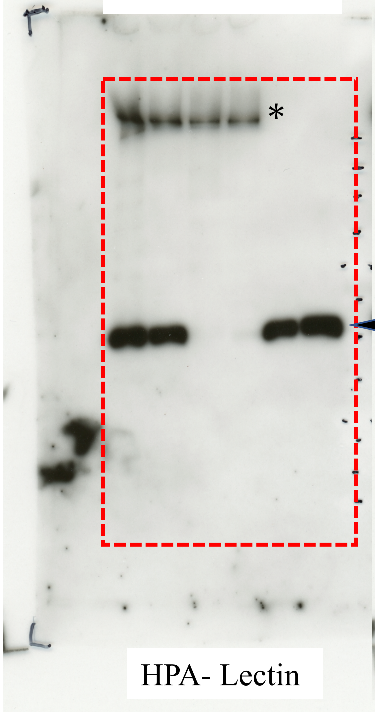

Fig4A lower panel

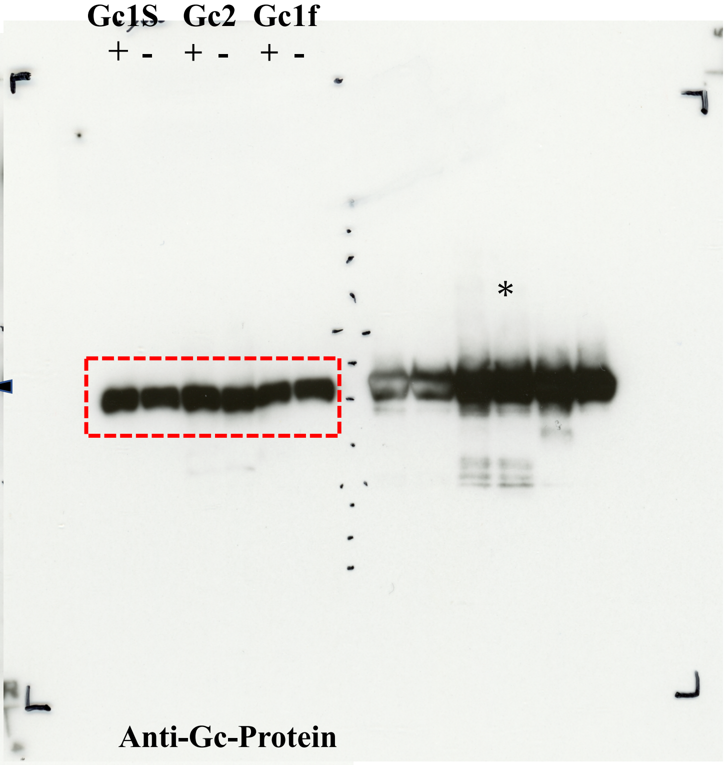

Full length blots of Fig. 4(B)

Fig.4B

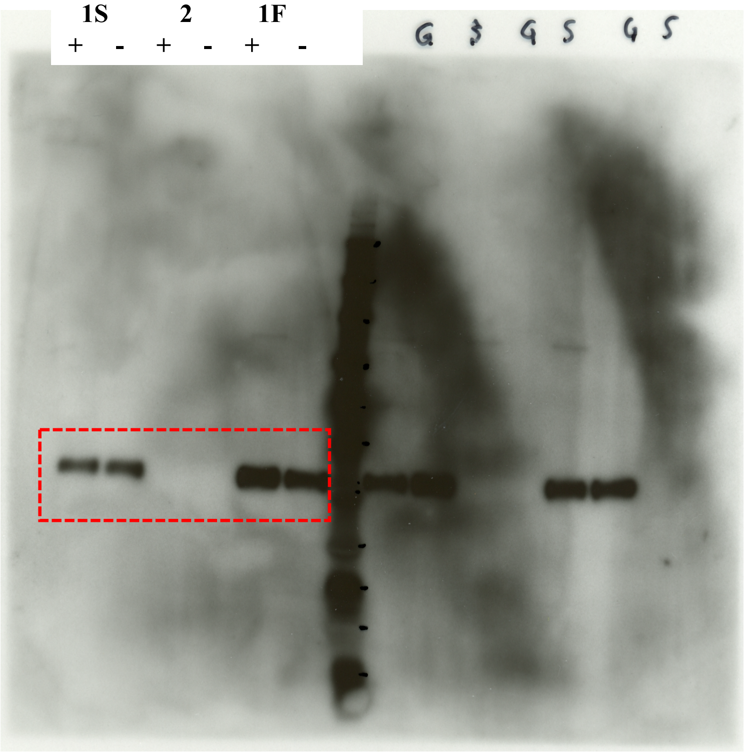

HPA

Full length blots of Fig. 4(B)

Fig.4B

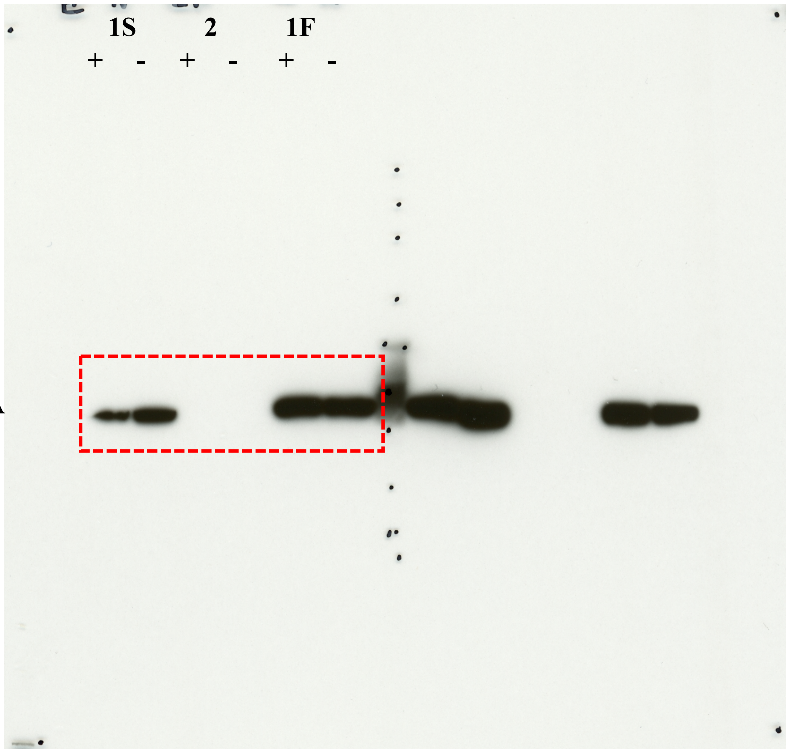

VVA

Full length blots of Fig. 4(B)

Fig.4B

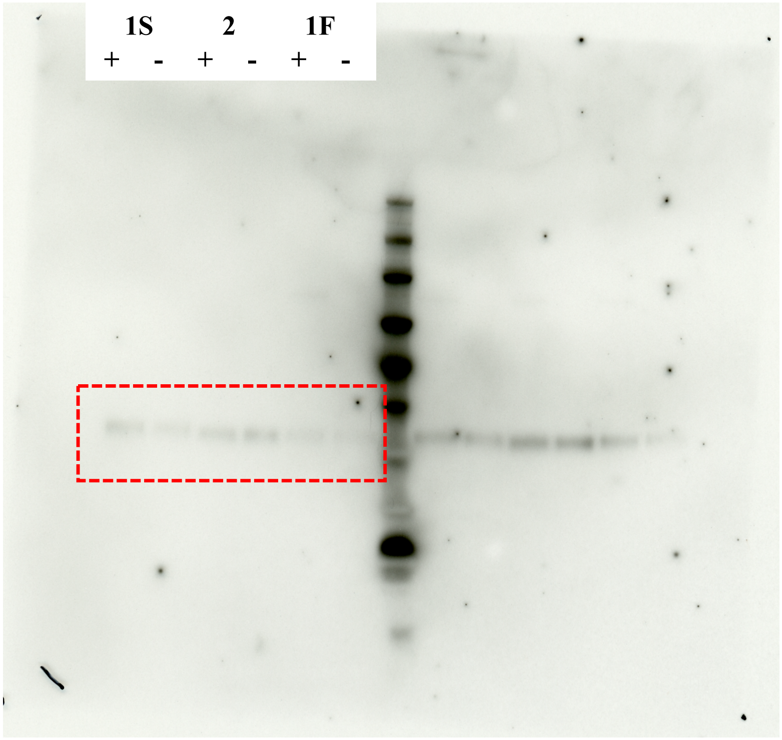

PNA

Full length blots of Fig. 4(B)

Fid.4B

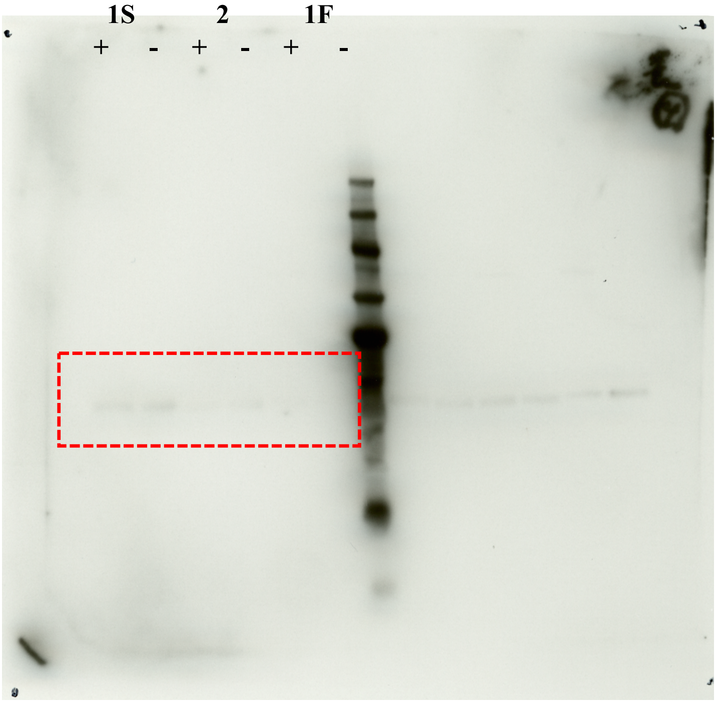

MALII

Full length blots of Fig. 5(A) and 5)B)

Fig.5A,B

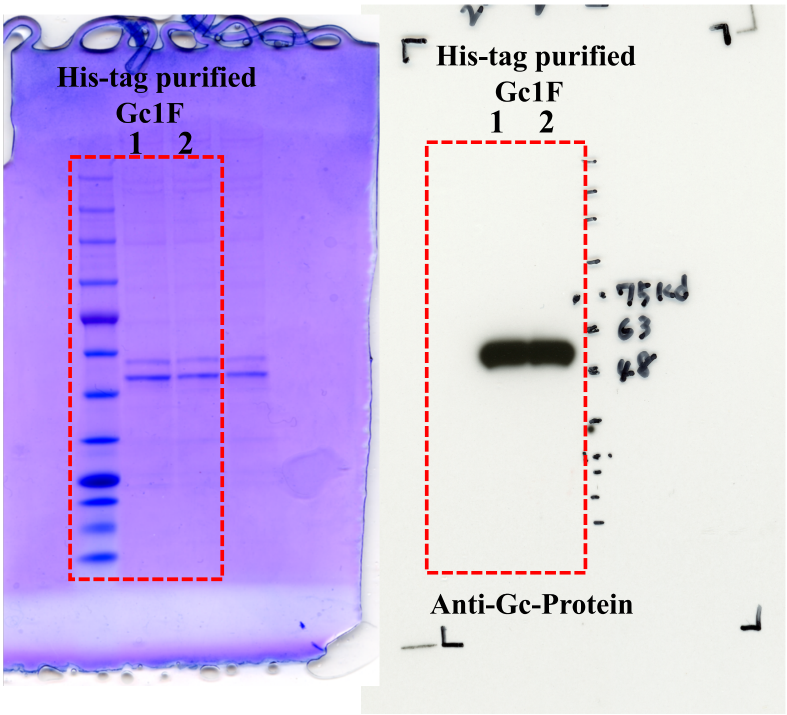

Full length blots of Fig. 5(C), 5(D) and 5(E)

Fig5 C,D,E

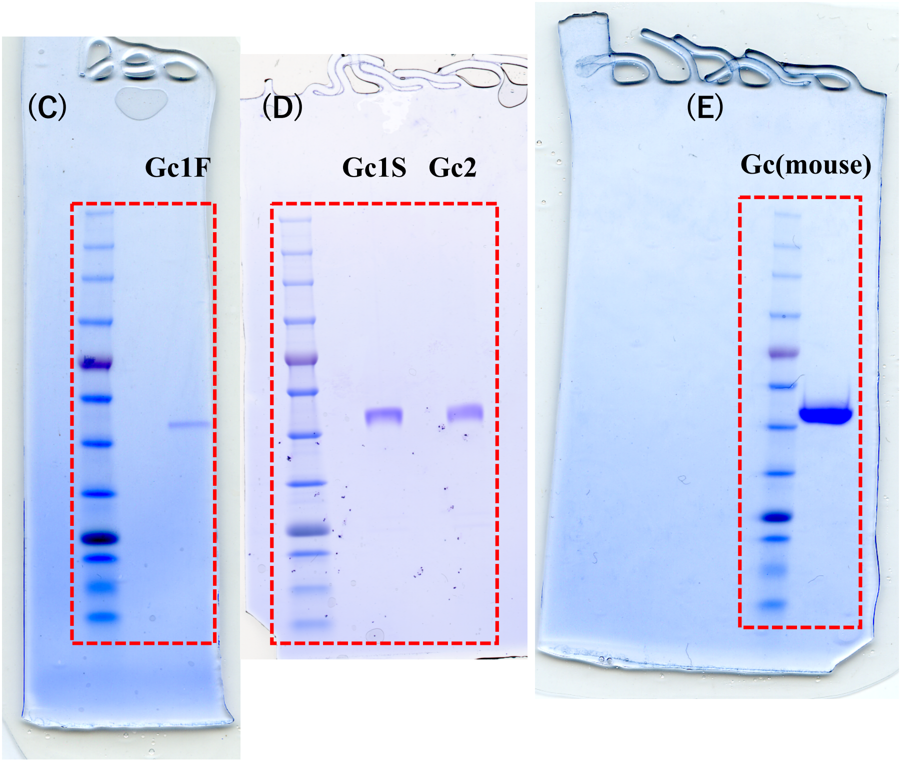

Supplementary Table 1

(A) Phagocytosis assay：GcMAF subtype (1f, 1s, 2)

	Phagocytosis index	Phagocytosi index		Fold value
		Ave	SD	vs control
Control No.1	3.15	3.67	0.47	1.00
Control No.1	3.79			
Control No.1	4.06			
LPS No.1	6.31	6.92	1.51	1.88
LPS No.2	5.80			
LPS No.3	8.64			
GcMAF 1f 10 ng No.1	7.63	7.18	1.09	1.96
GcMAF 1f 10 ng No.2	5.94			
GcMAF 1f 10 ng No.3	7.98			
GcMAF 1s 10 ng No.1	7.53	6.49	1.00	1.77
GcMAF 1s 10 ng No.2	6.42			
GcMAF 1s 10 ng No.3	5.52			
GcMAF 2 10 ng No.1	5.92	5.70	0.31	1.55
GcMAF 2 10 ng No.2	5.85			
GcMAF 2 10 ng No.3	5.34			

(B) Phagocytosis assay：Eenzymatically treated GcMAF

	Phagocytosis index	Phagocytosi index		Fold value
		Ave	SD	vs control
Control ①	2.02	1.93	0.15	1.00
Control ②	2.01			
Control ③	1.75			
GcMAF 1f 10 ng Expi CHO ①	2.67	2.80	0.12	1.45
GcMAF 1f 10 ng Expi CHO ②	2.90			
GcMAF 1f 10 ng Expi CHO ③	2.84			
GcMAF 1f 10 ng_CHO VD column ①	2.21	2.16	0.14	1.12
GcMAF 1f 10 ng_CHO VD column ②	2.27			
GcMAF 1f 10 ng_CHO VD column ③	2.01			
GcMAF 1f 10 ng_CHO VD column【Enzyme treatment】①	2.83	2.75	0.28	1.43
GcMAF 1f 10 ng_CHO VD column【Enzyme treatment】②	2.44			
GcMAF 1f 10 ng_CHO VD column【Enzyme treatment】③	2.99			

(C) Phagocytosis assay：GcMAF 1f dose dependent

	Phagocytosis index	Phagocytosi index		Fold value
		Ave	SD	vs control
Control ①	2.07	2.98	0.79	1.00
Control ②	3.33			
Control ③	3.53			
GcMAF 1f 0.1 ng ①	3.57	3.62	0.51	1.22
GcMAF 1f 0.1 ng ②	3.14			
GcMAF 1f 0.1 ng ③	4.16			
GcMAF 1f 1 ng ①	4.32	3.99	0.54	1.34
GcMAF 1f 1 ng ②	4.29			
GcMAF 1f 1 ng ③	3.38			
GcMAF 1f 10 ng ①	4.11	4.25	0.12	1.43
GcMAF 1f 10 ng ②	4.33			
GcMAF 1f 10 ng ③	4.30			
GcMAF 1f 100 ng ①	6.73	5.32	1.23	1.79
GcMAF 1f 100 ng ②	4.43			
GcMAF 1f 100 ng ③	4.81			

(C)Phagocytosis assay：GcMAF 1s dose dependent

	Phagocytosis index	Phagocytosi index		Fold value
		Ave	SD	vs control
Control ①	2.34	2.40	0.13	1.00
Control ②	2.31			
Control ③	2.55			
GcMAF 1s 0.1 ng ①	2.57	2.74	0.22	1.14
GcMAF 1s 0.1 ng ②	3.00			
GcMAF 1s 0.1 ng ③	2.66			
GcMAF 1s 1 ng ①	2.80	3.21	0.35	1.34
GcMAF 1s 1 ng ②	3.36			
GcMAF 1s 1 ng ③	3.45			
GcMAF 1s 10 ng ①	3.54	3.53	0.22	1.47
GcMAF 1s 10 ng ②	3.31			
GcMAF 1s 10 ng ③	3.75			
GcMAF 1s 100 ng ①	3.86	4.04	0.19	1.69
GcMAF 1s 100 ng ②	4.23			
GcMAF 1s 100 ng ③	4.03			

(C)Phagocytosis assay：GcMAF 2 dose dependent

	Phagocytosis index	Phagocytosi index		Fold value
		Ave	SD	vs control
Control ①	1.82	2.03	0.27	1.00
Control ②	2.34			
Control ③	1.94			
GcMAF 2 0.1 ng ①	2.28	2.08	0.26	1.02
GcMAF 2 0.1 ng ②	1.79			
GcMAF 2 0.1 ng ③	2.18			
GcMAF 2 1 ng ①	2.38	2.45	0.08	1.25
GcMAF 2 1 ng ②	2.54			
GcMAF 2 1 ng ③	2.43			
GcMAF 2 10 ng ①	2.83	2.83	0.15	1.32
GcMAF 2 10 ng ②	2.98			
GcMAF 2 10 ng ③	2.69			
GcMAF 2 100 ng ①	3.06	3.13	0.09	1.56
GcMAF 2 100 ng ②	3.10			
GcMAF 2 100 ng ③	3.23			
